# Supplementary material for: Feasibility, Effectiveness and Safety of Elastomeric Pumps for Delivery of Antibiotics to Adult Hospital Inpatients—A Systematic Review
Source: Antibiotics (Basel). 2023 Aug 22;12(9):1351. doi: 10.3390/antibiotics12091351 (PMC10525832; doi:10.3390/antibiotics12091351)
Supplement: Supplementary file 1 [file antibiotics-12-01351-s001.zip › Supplementary Materials.pdf]

Supplementary materials

Table S1 – AMED search strategy and results
17th December 2022

	Search terms	Hits
1	Elastomeric pump*.mp.	2
2	Balloon pump*.mp.	2
3	Ball pump*.mp. [mp=abstract, heading words, title]	0
4	Portable pump*.mp.	0
5	Accufuser*.mp. [mp=abstract, heading words, title]	0
6	Lv10*.mp. [mp=abstract, heading words, title]	0
7	Intimate*.mp.	281
8	Infusor*.mp.	2
9	1 or 2 or 3 or 4 or 5 or 6 or 7 or 8	287
10	Antiinfective agent*.mp.	1051
11	Exp antiinfective agents/	4963
12	Infectious agent*.mp.	28
13	Exp antibacterial agents/	1766
14	Exp antifungal agents/	690
15	Exp antiviral agents/	754
16	10 or 11 or 12 or 13 or 14 or 15	4985
17	(amikacin* or amoxicillin* or amoxycillin* or ampicillin* or azithromycin* or aztreonam* or bleomycin* or carbenicillin* or cefazolin* or cefepime*).mp. [mp=abstract, heading words, title]	164
18	(cefiderocol* or cefmetazole* or cefonicid* or cefoperazone* or cefotaxime* or cefotetan* or cefoxitin* or ceftaroline* or ceftazidime* or ceftazidimeavibactam*).mp. [mp=abstract, heading words, title]	11
19	(ceftazidime-avibactam* or ceftizoxime* or ceftolozanetazobactam* or ceftolozane-tazobactam* or ceftriaxone* or cefuroxime* or cephalothin* or cephapirin* or chloramphenicol* or ciprofloxacin*).mp. [mp=abstract, heading words, title]	99
20	(clarithromycin* or clindamycin* or cloxacillin* or colistimethate* or dactinomycin* or daptomycin* or delafloxacin* or doripenem* or eravacycline* or ertapenem*).mp. [mp=abstract, heading words, title]	40
21	(erythromycin* or flucloxacillin* or fosfomycin* or fusidic* or gentamicin* or imipenem* or imipenem-cilastatin-relebactam* or kanamycin* or lefamulin* or levofloxacin*).mp. [mp=abstract, heading words, title]	111
22	(lincomycin* or linezolid* or meropenem* or meropenemvaborbactam* or methicillin* or metronidazole* or mezlocillin* or mitomycin* or moxifloxacin* or nafcillin*).mp. [mp=abstract, heading words, title]	348
23	(netilmicin* or oritavancin* or oxacillin* or oxytetracycline* or penicillin* or piperacillin* or piperacillin-tazobactam* or piperacillintazobactam* or plazomicin* or plicamycin* or polymyxin*).mp. [mp=abstract, heading words, title]	101
24	(quinupristin-dalfopristin* or sulfamethoxazole* or sulfisoxazole* or teicoplanin* or telavancin* or temocillin* or ticarcillin* or tigecycline* or tobramycin* or trovafloxacin* or vancomycin*).mp. [mp=abstract, heading words, title]	84
25	(aciclovir* or acyclovir* or amphotericin* or liposomal-amphotericin* or ambisome* or anidulafungin* or caspofungin* or eravacycline* or famciclovir* or fluconazole*).mp. [mp=abstract, heading words, title]	153

26	(flucytosine* or foscarnet* or ganciclovir* or isavuconazonium* or isoconazole* or micafungin* or pentamidine* or rifampin* or voriconazole*).mp. [mp=abstract, heading words, title]	36
27	17 or 18 or 19 or 20 or 21 or 22 or 23 or 24 or 25 or 26	947
28	16 or 27	5438
29	Intravenous*.mp.	933
30	Exp drug administration routes/	1380
31	Parenteral*.mp.	241
32	29 or 30 or 31	2442
33	28 and 32	73
34	Limit 33 to" yr =2021-2022"	1

Table S2 – CINHAL search strategy and results
31st December 22

	Search Terms	Hits
1	(mh "infusion pumps+")	6,418
2	(mh "elastomers+")	3,323
3	S1 or s2	9,716
4	Elastomeric pump*	85
5	Balloon pump*	1,554
6	Ball pump*	0
7	Portable pump*	54
8	Acufuser*	3
9	Lv10*	5
10	Intimate*	21,178
11	Infusor*	1,223
12	Mh "drug delivery systems+")	10,412
13	S4 or s5 or s6 or s7 or s8 or s9 or s10 or s11 or s12	34,401
14	S3 or s13	39,512
15	(mh "antiinfective agents+")	181,256
16	(mh "antibiotics, antifungal+")	2,125
17	(mh "antifungal agents+")	9,917
18	Amikacin* or amoxicillin* or amoxycillin* or ampicillin* or azithromycin* or aztreonam* or bleomycin* or carbenicillin* or cefazolin* or cefepime*	13,716
19	Cefiderocol* or cefmetazole* or cefonicid* or cefoperazone* or cefotaxime* or cefotetan* or cefoxitin* or ceftaroline* or ceftazidime* or ceftazidimeavibactam*	3,511
20	Ceftazidime-avibactam* or ceftizoxime* or ceftolozanetazobactam* or ceftolozane-tazobactam* or ceftriaxone* or cefuroxime* or cephalothin* or cephapirin* or chloramphenicol* or ciprofloxacin*	8,235
21	Clarithromycin* or clindamycin* or cloxacillin* or colistimethate* or dactinomycin* or daptomycin* or delafloxacin* or doripenem* or eravacycline* or ertapenem*	5,812
22	Erythromycin* or flucloxacillin* or fosfomycin* or fusidic* or gentamicin* or imipenem* or imipenem-cilastatin-relebactam* or kanamycin* or lefamulin* or levofloxacin*	8,624

23	Lincomycin* or linezolid* or meropenem* or meropenemvaborbactam* or methicillin* or metronidazole* or mezlocillin* or mitomycin* or moxifloxacin* or nafcillin*	20,481
24	Netilmicin* or oritavancin* or oxacillin* or oxytetracycline* or penicillin* or piperacillin* or piperacillin-tazobactam* or piperacillintazobactam* or plazomicin* or plicamycin* or polymyxin*	8,455
25	Quinupristin-dalfopristin* or sulfamethoxazole* or sulfisoxazole* or teicoplanin* or telavancin* or temocillin* or ticarcillin* or tigecycline* or tobramycin* or trovafloxacin* or vancomycin*	11,751
26	Aciclovir* or acyclovir* or amphotericin* or liposomal-amphotericin* or ambisome* or anidulafungin* or caspofungin* or eravacycline* or famciclovir* or fluconazole*	7,091
27	Flucytosine* or foscarnet* or ganciclovir* or isavuconazonium* or isoconazole* or micafungin* or pentamidine* or rifampin* or voriconazole*	5,689
28	S15 or s16 or s17 or s18 or s19 or s20 or s21 or s22 or s23 or s24 or s25 or s26 or s27	200,970
29	S14 and s28	1,580
30	(mh "infusions, intravenous")	11,721
31	(mh "intravenous therapy+")	13,577
32	(mh "infusions, parenteral+")	16,463
33	Infusion* or intravenous* or parenteral*	132,114
34	S30 or s31 or s32 or s33	133,487
35	S29 and s34	310
36	S29 and s34 limiters – published date 2021010 - 20221231	19

Table S3 – Cochrane library search strategy and results 5th February 2023

	Search Terms	Hits
1	Elastomer* Elastomeric pump* or balloon pump* or ball pump* or portable pump* or accufuser* or lv10* or intimate* or infusor* in all text	3063
2	Antiinfective agent* or anti-infective* or infectious agent* or antibiotic*or antifungal agent* or antivirus agent* or amikacin* or amoxicillin* or amoxycillin* or ampicillin* or azithromycin* or aztreonam* or bleomycin* or carbenicillin* or cefazolin* or cefepimecefiderocol* or cefmetazole* or cefonicid* or cefoperazone* or cefotaxime* or cefotetan* or cefoxitin* or ceftaroline* or ceftazidime* or ceftazidimeavibactam*ceftazidime-avibactam* or ceftizoxime* or ceftolozanetazobactam* or ceftolozane-tazobactam* or ceftriaxone* or cefuroxime* or cephalothin* or cephapirin* or chloramphenicol* or ciprofloxacin*clarithromycin* or clindamycin* or cloxacillin* or colistimethate* or dactinomycin* or daptomycin* or delafloxacin* or doripenem* or eravacycline* or ertapenem*erythromycin* or flucloxacillin* or fosfomycin* or fusidic* or gentamicin* or imipenem* or imipenem-cilastatin-relebactam* or kanamycin* or lefamulin* or levofloxacin*lincomycin* or linezolid* or meropenem* or meropenemvaborbactam* or methicillin* or metronidazole* or mezlocillin* or mitomycin* or moxifloxacin* or nafcillin*netilmicin* or oritavancin* or oxacillin* or oxytetracycline* or penicillin* or piperacillin* or piperacillin-tazobactam* or piperacillintazobactam* or plazomicin* or plicamycin* or polymyxin*quinupristin-dalfopristin* or sulfamethoxazole* or sulfisoxazole* or	55170

	teicoplanin* or telavancin* or temocillin* or ticarcillin* or tigecycline* or tobramycin* or trovafloxacin* or vancomycin*aciclovir* or acyclovir* or amphotericin* or liposomal-amphotericin* or ambisome* or anidulafungin* or caspofungin* or eravacycline* or famciclovir* or fluconazole*flucytosine* or foscarnet* or ganciclovir* or isavuconazonium* or isoconazole* or micafungin* or pentamidine* or rifampin* or voriconazole* in all text- (word variations have been searched)	
3	1 and 2	58
4	3 Limited to 2021 – 2022	10

Table S4 – EMBASE search strategy and results 17th December 2022

	Search terms	Hits
1	Exp elastomeric infusion pump/	128
2	Exp elastomer/	5323
3	Exp infusion pump/	12022
4	Exp balloon pump/	8275
5	Exp elastomeric pump/	128
6	1 or 2 or 3 or 4 or 5	25415
7	Elastomeric pump*.mp.	416
8	Balloon pump*.mp.	12633
9	Ball pump*.mp.	2
10	Portable pump*.mp.	447
11	Accufuser*.mp.	56
12	Lv10*.mp.	62
13	Intimate*.mp.	49000
14	Infusor*.mp.	1917
15	Exp drug delivery system/	413092
16	Exp drug delivery device/	117708
17	15 or 16	413092
18	7 or 8 or 9 or 10 or 11 or 12 or 13 or 14	64412
19	6 or 18	81254
20	17 or 19	480875
21	Exp antiinfective agent/	4377489
22	Exp anti-infective therapy/	278552
23	Exp infectious agent/	34099
24	Exp antibiotic therapy/	147871
25	Exp antifungal agent/	417658
26	Exp antivirus agent/	1366712
27	21 or 22 or 23 or 24 or 25 or 26	4449170
28	(amikacin* or amoxicillin* or amoxycillin* or ampicillin* or azithromycin* or aztreonam* or bleomycin* or carbenicillin* or cefazolin* or cefepime*).mp. [mp=title, abstract, heading word, drug trade name, original title, device manufacturer, drug manufacturer, device trade name, keyword heading word, floating subheading word, candidate term word]	357840
29	(cefiderocol* or cefmetazole* or cefonicid* or cefoperazone* or cefotaxime* or cefotetan* or cefoxitin* or ceftaroline* or ceftazidime* or ceftazidimeavibactam*).mp. [mp=title, abstract, heading word, drug trade name,	107362

	original title, device manufacturer, drug manufacturer, device trade name, keyword heading word, floating subheading word, candidate term word]	
30	(ceftazidime-avibactam* or ceftizoxime* or ceftolozanetazobactam* or ceftolozane-tazobactam* or ceftriaxone* or cefuroxime* or cephalothin* or cephapirin* or chloramphenicol* or ciprofloxacin*).mp. [mp=title, abstract, heading word, drug trade name, original title, device manufacturer, drug manufacturer, device trade name, keyword heading word, floating subheading word, candidate term word]	255009
31	(clarithromycin* or clindamycin* or cloxacillin* or colistimethate* or dactinomycin* or daptomycin* or delafloxacin* or doripenem* or eravacycline* or ertapenem*).mp. [mp=title, abstract, heading word, drug trade name, original title, device manufacturer, drug manufacturer, device trade name, keyword heading word, floating subheading word, candidate term word]	161637
32	(erythromycin* or flucloxacillin* or fosfomycin* or fusidic* or gentamicin* or imipenem* or imipenem-cilastatin-relebactam* or kanamycin* or lefamulin* or levofloxacin*).mp. [mp=title, abstract, heading word, drug trade name, original title, device manufacturer, drug manufacturer, device trade name, keyword heading word, floating subheading word, candidate term word]	282738
33	(lincomycin* or linezolid* or meropenem* or meropenemvaborbactam* or methicillin* or metronidazole* or mezlocillin* or mitomycin* or moxifloxacin* or nafcillin*).mp. [mp=title, abstract, heading word, drug trade name, original title, device manufacturer, drug manufacturer, device trade name, keyword heading word, floating subheading word, candidate term word]	289090
34	(netilmicin* or oritavancin* or oxacillin* or oxytetracycline* or penicillin* or piperacillin* or piperacillin-tazobactam* or piperacillintazobactam* or plazomicin* or plicamycin* or polymyxin*).mp. [mp=title, abstract, heading word, drug trade name, original title, device manufacturer, drug manufacturer, device trade name, keyword heading word, floating subheading word, candidate term word]	246419
35	(quinupristin-dalfopristin* or sulfamethoxazole* or sulfisoxazole* or teicoplanin* or telavancin* or temocillin* or ticarcillin* or tigecycline* or tobramycin* or trovafloxacin* or vancomycin*).mp. [mp=title, abstract, heading word, drug trade name, original title, device manufacturer, drug manufacturer, device trade name, keyword heading word, floating subheading word, candidate term word]	198612
36	(aciclovir* or acyclovir* or amphotericin* or liposomal-amphotericin* or ambisome* or anidulafungin* or caspofungin* or eravacycline* or famciclovir* or fluconazole*).mp. [mp=title, abstract, heading word, drug trade name, original title, device manufacturer, drug manufacturer, device trade name, keyword heading word, floating subheading word, candidate term word]	144975
37	(flucytosine* or foscarnet* or ganciclovir* or isavuconazonium* or isoconazole* or micafungin* or pentamidine* or rifampin* or voriconazole*).mp. [mp=title, abstract, heading word, drug trade name, original title, device manufacturer, drug manufacturer, device trade name, keyword heading word, floating subheading word, candidate term word]	90285
38	29 or 30 or 31 or 32 or 33 or 34 or 35 or 36 or 37	976752
39	27 or 38	4541224
40	Exp continuous infusion/	49131
41	Exp drug infusion/	16515
42	20 and 39	104207
43	Infusion*.mp.	445933
44	40 or 41 or 43	445933

45	42 and 44	3887
46	Exp intravenous drug administration/	372388
47	Exp parenteral drug administration/	760458
48	(parenteral* or intravenous*).mp. [mp=title, abstract, heading word, drug trade name, original title, device manufacturer, drug manufacturer, device trade name, keyword heading word, floating subheading word, candidate term word]	1339501
49	46 or 47 or 48	1712002
50	45 and 49	2116
51	Limit 50 to "yr 2021- current"	286

Table S5 – OVID MEDLINE search stratergy and results 17th November 2021

	Search Terms	Hits
1	Exp infusion pumps/	15225
2	Exp elastomers/	37850
3	1 or 2	52957
4	Elastomeric pump*.mp.	211
5	Balloon pump*.mp.	7354
6	Ball pump*.mp.	3
7	Portable pump*.mp.	332
8	Accufuser*.mp.	5
9	Lv10*.mp.	25
10	Intimate*.mp.	43460
11	Infusor*.mp.	501
12	Exp drug delivery systems/	167637
13	4 or 5 or 6 or 7 or 8 or 9 or 10 or 11 or 12	219274
14	3 or 13	264527
15	Exp anti-infective agents/	1796567
16	Exp anti-bacterial agents/	805443
17	Exp antifungal agents/	184427
18	15 or 16 or 17	1796567
19	(amikacin* or amoxicillin* or amoxycillin* or ampicillin* or azithromycin* or aztreonam* or bleomycin* or carbenicillin* or cefazolin* or cefepime*).mp. [mp=title, abstract, original title, name of substance word, subject heading word, floating sub-heading word, keyword heading word, organism supplementary concept word, protocol supplementary concept word, rare disease supplementary concept word, unique identifier, synonyms]	108600
20	(cefiderocol* or cefmetazole* or cefonicid* or cefoperazone* or cefotaxime* or cefotetan* or cefoxitin* or ceftaroline* or ceftazidime* or ceftazidimeavibactam*).mp. [mp=title, abstract, original title, name of substance word, subject heading word, floating sub-heading word, keyword heading word, organism supplementary concept word, protocol supplementary concept word, rare disease supplementary concept word, unique identifier, synonyms]	29318
21	(clarithromycin* or clindamycin* or cloxacillin* or colistimethate* or dactinomycin* or daptomycin* or delafloxacin* or doripenem* or eravacycline* or ertapenem*).mp. [mp=title, abstract, original title, name of substance word, subject heading word, floating sub-heading word, keyword heading word, organism supplementary concept word, protocol supplementary concept word, rare disease supplementary concept word, unique identifier, synonyms]	54341

22	(erythromycin* or flucloxacillin* or fosfomycin* or fusidic* or gentamicin* or imipenem* or imipenem-cilastatin-relebactam* or kanamycin* or lefamulin* or levofloxacin*).mp. [mp=title, abstract, original title, name of substance word, subject heading word, floating sub-heading word, keyword heading word, organism supplementary concept word, protocol supplementary concept word, rare disease supplementary concept word, unique identifier, synonyms]	95838
23	(lincomycin* or linezolid* or meropenem* or meropenemvaborbactam* or methicillin* or metronidazole* or mezlocillin* or mitomycin* or moxifloxacin* or nafcillin*).mp. [mp=title, abstract, original title, name of substance word, subject heading word, floating sub-heading word, keyword heading word, organism supplementary concept word, protocol supplementary concept word, rare disease supplementary concept word, unique identifier, synonyms]	115820
24	(netilmicin* or oritavancin* or oxacillin* or oxytetracycline* or penicillin* or piperacillin* or piperacillin-tazobactam* or piperacillintazobactam* or plazomicin* or plicamycin* or polymyxin*).mp. [mp=title, abstract, original title, name of substance word, subject heading word, floating sub-heading word, keyword heading word, organism supplementary concept word, protocol supplementary concept word, rare disease supplementary concept word, unique identifier, synonyms]	121111
25	(quinupristin-dalfopristin* or sulfamethoxazole* or sulfisoxazole* or teicoplanin* or telavancin* or temocillin* or ticarcillin* or tigecycline* or tobramycin* or trovafloxacin* or vancomycin*).mp. [mp=title, abstract, original title, name of substance word, subject heading word, floating sub-heading word, keyword heading word, organism supplementary concept word, protocol supplementary concept word, rare disease supplementary concept word, unique identifier, synonyms]	73785
26	(aciclovir* or acyclovir* or amphotericin* or liposomal-amphotericin* or ambisome* or anidulafungin* or caspofungin* or eravacycline* or famciclovir* or fluconazole*).mp. [mp=title, abstract, original title, name of substance word, subject heading word, floating sub-heading word, keyword heading word, organism supplementary concept word, protocol supplementary concept word, rare disease supplementary concept word, unique identifier, synonyms]	52861
27	(flucytosine* or foscarnet* or ganciclovir* or isavuconazonium* or isoconazole* or micafungin* or pentamidine* or rifampin* or voriconazole*).mp. [mp=title, abstract, original title, name of substance word, subject heading word, floating sub-heading word, keyword heading word, organism supplementary concept word, protocol supplementary concept word, rare disease supplementary concept word, unique identifier, synonyms]	50774
28	19 or 20 or 21 or 22 or 23 or 24 or 25 or 26 or 27	505350
29	18 or 28	1937526
30	14 and 29	26147
31	Exp infusions, intravenous/	56872
32	Infusion*.mp.	316522
33	Continuous infusion*.mp.	19793
34	Exp infusions, parenteral/	95128
35	Parenteral*.mp.	91905
36	Exp administration, intravenous/	148447
37	31 or 32 or 33 or 34 or 35 or 36	457221
38	30 and 37	1564
39	Limit 38 to "yr 2021-2022"	43

Table S6 – Pubmed search strategy and results 31st December 2022

	Search Terms	Hits
1	elastomer[mesh terms]	37841
2	infusion pumps [mesh terms]	15216
3	external infusion pump[mesh terms]	15216
4	Elastomeric-pump* or balloon-pump* or ball-pump* or portable pump* or accufuser* or lv10*,most recent,,""""elastomeric pump*""[all fields] or ""balloon pump*""[all fields] or ""ball pump*""[all fields] or ((""portability""[all fields] or ""portable""[all fields] or ""portables""[all fields]) and ""pump*""[all fields]) or ""accufuser*""[all fields] or ""lv10*""[all fields]"	9299
5	drug delivery system[mesh terms]	165722
6	((((drug delivery system[mesh terms]) or (elastomeric-pump* or balloon-pump* or ball-pump* or portable pump* or accufuser* or lv10*)) or (external infusion pump[mesh terms])) or (infusion pump[mesh terms])) or (elastomer[mesh terms]),most recent,,""""drug delivery systems""[mesh terms] or (""elastomeric pump*""[all fields] or ""balloon pump*""[all fields] or ""ball pump*""[all fields] or ((""portability""[all fields] or ""portable""[all fields] or ""portables""[all fields]) and ""pump*""[all fields]) or ""accufuser*""[all fields] or ""lv10*""[all fields]) or ""infusion pumps""[mesh terms] or ""infusion pumps""[mesh terms] or ""elastomers""[mesh terms]"	220034
7	amikacin* or amoxicillin* or amoxycillin* or ampicillin* or azithromycin* or aztreonam* or bleomycin* or carbenicillin* or cefazolin* or cefepime* or cefiderocol* or cefmetazole* or cefonicid* or cefoperazone* or cefotaxime* or cefotetan* or cefoxitin* or ceftaroline* or ceftazidime* or ceftazidimeavibactam* or ceftazidime-avibactam* or ceftizoxime* or ceftolozanetazobactam* or ceftolozane-tazobactam* or ceftriaxone* or cefuroxime* or cephalothin* or cephapirin* or chloramphenicol* or ciprofloxacin* or clarithromycin* or clindamycin* or cloxacillin* or colistimethate* or dactinomycin* or daptomycin* or delafloxacin* or doripenem* or eravacycline* or ertapenem* or erythromycin* or flucloxacillin* or fosfomycin* or fusidic* or gentamicin* or imipenem* or imipenem-cilastatin-relebactam* or kanamycin* or lefamulin* or levofloxacin* or lincomycin* or linezolid* or meropenem* or meropenemvaborbactam* or methicillin* or metronidazole* or mezlocillin* or mitomycin* or moxifloxacin* or nafcillin* or netilmicin* or oritavancin* or oxacillin* or oxytetracycline* or penicillin* or piperacillin* or piperacillin-tazobactam* or piperacillintazobactam* or plazomicin* or plicamycin* or polymyxin* or quinupristin-dalfopristin* or sulfamethoxazole* or sulfisoxazole* or teicoplanin* or telavancin* or temocillin* or ticarcillin* or tigecycline* or tobramycin* or trovafloxacin* or vancomycin* or aciclovir* or acyclovir* or amphotericin* or liposomal-amphotericin* or ambisome* or anidulafungin* or caspofungin* or eravacycline* or famciclovir* or fluconazole* or flucytosine* or foscarnet* or ganciclovir* or isavuconazonium* or isoconazole* or micafungin* or pentamidine* or rifampin* or voriconazole*,most recent,,""""amikacin*""[all fields] or ""amoxicillin*""[all fields] or ""amoxycillin*""[all fields] or ""ampicillin*""[all fields] or ""azithromycin*""[all fields] or ""aztreonam*""[all fields] or ""bleomycin*""[all fields] or ""carbenicillin*""[all fields] or ""cefazolin*""[all fields] or ""cefepime*""[all fields] or ""cefiderocol*""[all fields] or ""cefmetazole*""[all fields] or ""cefonicid*""[all fields] or ""cefoperazone*""[all fields] or ""cefotaxime*""[all fields] or ""cefotetan*""[all fields] or ""cefoxitin*""[all fields] or ""ceftaroline*""[all fields] or	552445

	""ceftazidime*""[all fields] or ""ceftazidimeavibactam*""[all fields] or ""ceftazidime avibactam*""[all fields] or ""ceftizoxime*""[all fields] or ""ceftolozanetazobactam*""[all fields] or ""ceftolozane tazobactam*""[all fields] or ""ceftriaxone*""[all fields] or ""cefuroxime*""[all fields] or ""cephalothin*""[all fields] or ""cephapirin*""[all fields] or ""chloramphenicol*""[all fields] or ""ciprofloxacin*""[all fields] or ""clarithromycin*""[all fields] or ""clindamycin*""[all fields] or ""cloxacillin*""[all fields] or ""colistimethate*""[all fields] or ""dactinomycin*""[all fields] or ""daptomycin*""[all fields] or ""delafloxacin*""[all fields] or ""doripenem*""[all fields] or ""eravacycline*""[all fields] or ""ertapenem*""[all fields] or ""erythromycin*""[all fields] or ""flucloxacillin*""[all fields] or ""fosfomycin*""[all fields] or ""fusidic*""[all fields] or ""gentamicin*""[all fields] or ""imipenem*""[all fields] or ""imipenem cilastatin relebactam*""[all fields] or ""kanamycin*""[all fields] or ""lefamulin*""[all fields] or ""levofloxacin*""[all fields] or ""lincomycin*""[all fields] or ""linezolid*""[all fields] or ""meropenem*""[all fields] or ""meropenemvaborbactam*""[all fields] or ""methicillin*""[all fields] or ""metronidazole*""[all fields] or ""mezlocillin*""[all fields] or ""mitomycin*""[all fields] or ""moxifloxacin*""[all fields] or ""nafcillin*""[all fields] or ""netilmicin*""[all fields] or ""oritavancin*""[all fields] or ""oxacillin*""[all fields] or ""oxytetracycline*""[all fields] or ""penicillin*""[all fields] or ""piperacillin*""[all fields] or ""piperacillin tazobactam*""[all fields] or ""piperacillintazobactam*""[all fields] or ""plazomicin*""[all fields] or ""plicamycin*""[all fields] or ""polymyxin*""[all fields] or ""quinupristin dalfopristin*""[all fields] or ""sulfamethoxazole*""[all fields] or ""sulfisoxazole*""[all fields] or ""teicoplanin*""[all fields] or ""telavancin*""[all fields] or ""temocillin*""[all fields] or ""ticarcillin*""[all fields] or ""tigecycline*""[all fields] or ""tobramycin*""[all fields] or ""trovafloxacin*""[all fields] or ""vancomycin*""[all fields] or ""aciclovir*""[all fields] or ""acyclovir*""[all fields] or ""amphotericin*""[all fields] or ""liposomal amphotericin*""[all fields] or ""ambisome*""[all fields] or ""anidulafungin*""[all fields] or ""caspofungin*""[all fields] or ""eravacycline*""[all fields] or ""famciclovir*""[all fields] or ""fluconazole*""[all fields] or ""flucytosine*""[all fields] or ""foscarnet*""[all fields] or ""ganciclovir*""[all fields] or ""isavuconazonium*""[all fields] or ""isoconazole*""[all fields] or ""micafungin*""[all fields] or ""pentamidine*""[all fields] or ""rifampin*""[all fields] or ""voriconazole*""[all fields]"	
8	"agents, antiinfective [mesh terms]	815313
9	"anti-infective therapy" [mesh terms]	403921
10	antibiotic* or anti-biotic* or anti-bacterial* or antibacterial* or antimicrobial* or anti-microbial* or anti-fungal* or anti-viral* or antiviral* or infectious agent*,most recent,,"""antibiotic*""[all fields] or ""anti biotic*""[all fields] or ""anti bacterial*""[all fields] or ""antibacterial*""[all fields] or ""antimicrobial*""[all fields] or ""anti microbial*""[all fields] or ""anti fungal*""[all fields] or ""anti viral*""[all fields] or ""antiviral*""[all fields] or ((""infectious""[all fields] or ""infectiousness""[all fields]) and ""agent*""[all fields])"	1701309
11	(((amikacin* or amoxicillin* or amoxycillin* or ampicillin* or azithromycin* or aztreonam* or bleomycin* or carbenicillin* or cefazolin* or cefepime* or cefiderocol* or cefmetazole* or cefonicid* or cefoperazone* or cefotaxime* or cefotetan* or cefoxitin* or ceftaroline* or ceftazidime* or ceftazidimeavibactam* or ceftazidime-avibactam* or ceftizoxime* or ceftolozanetazobactam* or ceftolozane-tazobactam* or ceftriaxone* or cefuroxime* or cephalothin* or cephapirin* or chloramphenicol* or ciprofloxacin* or clarithromycin* or clindamycin* or cloxacillin* or colistimethate* or dactinomycin* or daptomycin* or delafloxacin* or	2036769

	doripenem* or eravacycline* or ertapenem* or erythromycin* or flucloxacillin* or fosfomycin* or fusidic* or gentamicin* or imipenem* or imipenem-cilastatin-relebactam* or kanamycin* or lefamulin* or levofloxacin* or lincomycin* or linezolid* or meropenem* or meropenemvaborbactam* or methicillin* or metronidazole* or mezlocillin* or mitomycin* or moxifloxacin* or nafcillin* or netilmicin* or oritavancin* or oxacillin* or oxytetracycline* or penicillin* or piperacillin* or piperacillin-tazobactam* or piperacillintazobactam* or plazomicin* or plicamycin* or polymyxin* or quinupristin-dalfopristin* or sulfamethoxazole* or sulfisoxazole* or teicoplanin* or telavancin* or temocillin* or ticarcillin* or tigecycline* or tobramycin* or trovafloxacin* or vancomycin* or aciclovir* or acyclovir* or amphotericin* or liposomal-amphotericin* or ambisome* or anidulafungin* or caspofungin* or eravacycline* or famciclovir* or fluconazole* or flucytosine* or foscarnet* or ganciclovir* or isavuconazonium* or isoconazole* or micafungin* or pentamidine* or rifampin* or voriconazole*) or (agents, antiinfective[mesh terms])) or (anti-infective therapy[mesh terms])) or (antibiotic* or anti-biotic* or anti-bacterial* or antibacterial* or antimicrobial* or anti-microbial* or anti-fungal* or anti-viral* or antiviral* or infectious agent*)",most recent,,"""amikacin*""[all fields] or ""amoxicillin*""[all fields] or ""amoxycillin*""[all fields] or ""ampicillin*""[all fields] or ""azithromycin*""[all fields] or ""aztreonam*""[all fields] or ""bleomycin*""[all fields] or ""carbenicillin*""[all fields] or ""cefazolin*""[all fields] or ""cefepime*""[all fields] or ""cefiderocol*""[all fields] or ""cefmetazole*""[all fields] or ""cefonicid*""[all fields] or ""cefoperazone*""[all fields] or ""cefotaxime*""[all fields] or ""cefotetan*""[all fields] or ""cefoxitin*""[all fields] or ""ceftaroline*""[all fields] or ""ceftazidime*""[all fields] or ""ceftazidimeavibactam*""[all fields] or ""ceftazidime avibactam*""[all fields] or ""ceftizoxime*""[all fields] or ""ceftolozanetazobactam*""[all fields] or ""ceftolozane tazobactam*""[all fields] or ""ceftriaxone*""[all fields] or ""cefuroxime*""[all fields] or ""cephalothin*""[all fields] or ""cephapirin*""[all fields] or ""chloramphenicol*""[all fields] or ""ciprofloxacin*""[all fields] or ""clarithromycin*""[all fields] or ""clindamycin*""[all fields] or ""cloxacillin*""[all fields] or ""colistimethate*""[all fields] or ""dactinomycin*""[all fields] or ""daptomycin*""[all fields] or ""delafloxacin*""[all fields] or ""doripenem*""[all fields] or ""eravacycline*""[all fields] or ""ertapenem*""[all fields] or ""erythromycin*""[all fields] or ""flucloxacillin*""[all fields] or ""fosfomycin*""[all fields] or ""fusidic*""[all fields] or ""gentamicin*""[all fields] or ""imipenem*""[all fields] or ""imipenem cilastatin relebactam*""[all fields] or ""kanamycin*""[all fields] or ""lefamulin*""[all fields] or ""levofloxacin*""[all fields] or ""lincomycin*""[all fields] or ""linezolid*""[all fields] or ""meropenem*""[all fields] or ""meropenemvaborbactam*""[all fields] or ""methicillin*""[all fields] or ""metronidazole*""[all fields] or ""mezlocillin*""[all fields] or ""mitomycin*""[all fields] or ""moxifloxacin*""[all fields] or ""nafcillin*""[all fields] or ""netilmicin*""[all fields] or ""oritavancin*""[all fields] or ""oxacillin*""[all fields] or ""oxytetracycline*""[all fields] or ""penicillin*""[all fields] or ""piperacillin*""[all fields] or ""piperacillin tazobactam*""[all fields] or ""piperacillintazobactam*""[all fields] or ""plazomicin*""[all fields] or ""plicamycin*""[all fields] or ""polymyxin*""[all fields] or ""quinupristin dalfopristin*""[all fields] or ""sulfamethoxazole*""[all fields] or ""sulfisoxazole*""[all fields] or ""teicoplanin*""[all fields] or ""telavancin*""[all fields] or ""temocillin*""[all fields] or ""ticarcillin*""[all fields] or ""tigecycline*""[all fields] or ""tobramycin*""[all fields] or ""trovafloxacin*""[all fields] or ""vancomycin*""[all fields] or ""aciclovir*""[all fields] or ""acyclovir*""[all fields] or	

	""amphotericin*""[all fields] or ""liposomal amphotericin*""[all fields] or ""ambisome*""[all fields] or ""anidulafungin*""[all fields] or ""caspofungin*""[all fields] or ""eravacycline*""[all fields] or ""famciclovir*""[all fields] or ""fluconazole*""[all fields] or ""flucytosine*""[all fields] or ""foscarnet*""[all fields] or ""ganciclovir*""[all fields] or ""isavuconazonium*""[all fields] or ""isoconazole*""[all fields] or ""micafungin*""[all fields] or ""pentamidine*""[all fields] or ""rifampin*""[all fields] or ""voriconazole*""[all fields] or ""anti infective agents""[mesh terms] or ((""anti infective agents""[pharmacological action] or ""anti infective agents""[mesh terms] or (""anti infective""[all fields] and ""agents""[all fields]) or ""anti infective agents""[all fields] or (""anti""[all fields] and ""infective""[all fields]) or ""anti infective""[all fields]) and ""therapeutics""[mesh terms]) or (""antibiotic*""[all fields] or ""anti biotic*""[all fields] or ""anti bacterial*""[all fields] or ""antibacterial*""[all fields] or ""antimicrobial*""[all fields] or ""anti microbial*""[all fields] or ""anti fungal*""[all fields] or ""anti viral*""[all fields] or ""antiviral*""[all fields] or ((""infectious""[all fields] or ""infectiousness""[all fields]) and ""agent*""[all fields]))"	
12	((((amikacin* or amoxicillin* or amoxycillin* or ampicillin* or azithromycin* or aztreonam* or bleomycin* or carbenicillin* or cefazolin* or cefepime* or cefiderocol* or cefmetazole* or cefonicid* or cefoperazone* or cefotaxime* or cefotetan* or cefoxitin* or ceftaroline* or ceftazidime* or ceftazidimeavibactam* or ceftazidime-avibactam* or ceftizoxime* or ceftolozanetazobactam* or ceftolozane-tazobactam* or ceftriaxone* or cefuroxime* or cephalothin* or cephapirin* or chloramphenicol* or ciprofloxacin* or clarithromycin* or clindamycin* or cloxacillin* or colistimethate* or dactinomycin* or daptomycin* or delafloxacin* or doripenem* or eravacycline* or ertapenem* or erythromycin* or flucloxacillin* or fosfomycin* or fusidic* or gentamicin* or imipenem* or imipenem-cilastatin-relebactam* or kanamycin* or lefamulin* or levofloxacin* or lincomycin* or linezolid* or meropenem* or meropenemvaborbactam* or methicillin* or metronidazole* or mezlocillin* or mitomycin* or moxifloxacin* or nafcillin* or netilmicin* or oritavancin* or oxacillin* or oxytetracycline* or penicillin* or piperacillin* or piperacillin-tazobactam* or piperacillintazobactam* or plazomicin* or plicamycin* or polymyxin* or quinupristin-dalfopristin* or sulfamethoxazole* or sulfisoxazole* or teicoplanin* or telavancin* or temocillin* or ticarcillin* or tigecycline* or tobramycin* or trovafloxacin* or vancomycin* or aciclovir* or acyclovir* or amphotericin* or liposomal-amphotericin* or ambisome* or anidulafungin* or caspofungin* or eravacycline* or famciclovir* or fluconazole* or flucytosine* or foscarnet* or ganciclovir* or isavuconazonium* or isoconazole* or micafungin* or pentamidine* or rifampin* or voriconazole*) or (agents, antiinfective[mesh terms])) or (anti-infective therapy[mesh terms])) or (antibiotic* or anti-biotic* or anti-bacterial* or antibacterial* or antimicrobial* or anti-microbial* or anti-fungal* or anti-viral* or antiviral* or infectious agent*)) and (((((drug delivery system[mesh terms]) or (elastomeric-pump* or balloon-pump* or ball-pump* or portable pump* or accufuser* or lv10*)) or (external infusion pump[mesh terms])) or (infusion pump[mesh terms])) or (elastomer[mesh terms]))",most recent,,"(""amikacin*""[all fields] or ""amoxicillin*""[all fields] or ""amoxycillin*""[all fields] or ""ampicillin*""[all fields] or ""azithromycin*""[all fields] or ""aztreonam*""[all fields] or ""bleomycin*""[all fields] or ""carbenicillin*""[all fields] or ""cefazolin*""[all fields] or ""cefepime*""[all fields] or ""cefiderocol*""[all fields] or ""cefmetazole*""[all fields] or ""cefonicid*""[all fields] or ""cefoperazone*""[all fields] or ""cefotaxime*""[all fields] or ""cefotetan*""[all fields] or ""cefoxitin*""[all fields] or ""ceftaroline*""[all fields] or	35810

	""ceftazidime*""[all fields] or ""ceftazidimeavibactam*""[all fields] or ""ceftazidime avibactam*""[all fields] or ""ceftizoxime*""[all fields] or ""ceftolozanetazobactam*""[all fields] or ""ceftolozane tazobactam*""[all fields] or ""ceftriaxone*""[all fields] or ""cefuroxime*""[all fields] or ""cephalothin*""[all fields] or ""cephapirin*""[all fields] or ""chloramphenicol*""[all fields] or ""ciprofloxacin*""[all fields] or ""clarithromycin*""[all fields] or ""clindamycin*""[all fields] or ""cloxacillin*""[all fields] or ""colistimethate*""[all fields] or ""dactinomycin*""[all fields] or ""daptomycin*""[all fields] or ""delafloxacin*""[all fields] or ""doripenem*""[all fields] or ""eravacycline*""[all fields] or ""ertapenem*""[all fields] or ""erythromycin*""[all fields] or ""flucloxacillin*""[all fields] or ""fosfomycin*""[all fields] or ""fusidic*""[all fields] or ""gentamicin*""[all fields] or ""imipenem*""[all fields] or ""imipenem cilastatin relebactam*""[all fields] or ""kanamycin*""[all fields] or ""lefamulin*""[all fields] or ""levofloxacin*""[all fields] or ""lincomycin*""[all fields] or ""linezolid*""[all fields] or ""meropenem*""[all fields] or ""meropenemvaborbactam*""[all fields] or ""methicillin*""[all fields] or ""metronidazole*""[all fields] or ""mezlocillin*""[all fields] or ""mitomycin*""[all fields] or ""moxifloxacin*""[all fields] or ""nafcillin*""[all fields] or ""netilmicin*""[all fields] or ""oritavancin*""[all fields] or ""oxacillin*""[all fields] or ""oxytetracycline*""[all fields] or ""penicillin*""[all fields] or ""piperacillin*""[all fields] or ""piperacillin tazobactam*""[all fields] or ""piperacillintazobactam*""[all fields] or ""plazomicin*""[all fields] or ""plicamycin*""[all fields] or ""polymyxin*""[all fields] or ""quinupristin dalfopristin*""[all fields] or ""sulfamethoxazole*""[all fields] or ""sulfisoxazole*""[all fields] or ""teicoplanin*""[all fields] or ""telavancin*""[all fields] or ""temocillin*""[all fields] or ""ticarcillin*""[all fields] or ""tigecycline*""[all fields] or ""tobramycin*""[all fields] or ""trovafloxacin*""[all fields] or ""vancomycin*""[all fields] or ""aciclovir*""[all fields] or ""acyclovir*""[all fields] or ""amphotericin*""[all fields] or ""liposomal amphotericin*""[all fields] or ""ambisome*""[all fields] or ""anidulafungin*""[all fields] or ""caspofungin*""[all fields] or ""eravacycline*""[all fields] or ""famciclovir*""[all fields] or ""fluconazole*""[all fields] or ""flucytosine*""[all fields] or ""foscarnet*""[all fields] or ""ganciclovir*""[all fields] or ""isavuconazonium*""[all fields] or ""isoconazole*""[all fields] or ""micafungin*""[all fields] or ""pentamidine*""[all fields] or ""rifampin*""[all fields] or ""voriconazole*""[all fields] or ""anti infective agents""[mesh terms] or ((""anti infective agents""[pharmacological action] or ""anti infective agents""[mesh terms] or (""anti infective""[all fields] and ""agents""[all fields]) or ""anti infective agents""[all fields] or (""anti""[all fields] and ""infective""[all fields]) or ""anti infective""[all fields]) and ""therapeutics""[mesh terms]) or (""antibiotic*""[all fields] or ""anti biotic*""[all fields] or ""anti bacterial*""[all fields] or ""antibacterial*""[all fields] or ""antimicrobial*""[all fields] or ""anti microbial*""[all fields] or ""anti fungal*""[all fields] or ""anti viral*""[all fields] or ""antiviral*""[all fields] or ((""infectious""[all fields] or ""infectiousness""[all fields]) and ""agent*""[all fields]))) and (""drug delivery systems""[mesh terms] or (""elastomeric pump*""[all fields] or ""balloon pump*""[all fields] or ""ball pump*""[all fields] or ((""portability""[all fields] or ""portable""[all fields] or ""portables""[all fields]) and ""pump*""[all fields]) or ""accufuser*""[all fields] or ""lv10*""[all fields]) or ""infusion pumps""[mesh terms] or ""infusion pumps""[mesh terms] or ""elastomers""[mesh terms])″	
13	administration, intravenous[mesh terms]	56867
14	Intravenous* or parenteral* [all fields]	554071

15	(administration, intravenous[mesh terms]) or (intravenous* or parenteral*)",most recent,,"""infusions, intravenous""[mesh terms] or ""intravenous*""[all fields] or ""parenteral*""[all fields]"	554071
16	"(((((amikacin* or amoxicillin* or amoxycillin* or ampicillin* or azithromycin* or aztreonam* or bleomycin* or carbenicillin* or cefazolin* or cefepime* or cefiderocol* or cefmetazole* or cefonicid* or cefoperazone* or cefotaxime* or cefotetan* or cefoxitin* or ceftaroline* or ceftazidime* or ceftazidimeavibactam* or ceftazidime-avibactam* or ceftizoxime* or ceftolozanetazobactam* or ceftolozane-tazobactam* or ceftriaxone* or cefuroxime* or cephalothin* or cephapirin* or chloramphenicol* or ciprofloxacin* or clarithromycin* or clindamycin* or cloxacillin* or colistimethate* or dactinomycin* or daptomycin* or delafloxacin* or doripenem* or eravacycline* or ertapenem* or erythromycin* or flucloxacillin* or fosfomycin* or fusidic* or gentamicin* or imipenem* or imipenem-cilastatin-relebactam* or kanamycin* or lefamulin* or levofloxacin* or lincomycin* or linezolid* or meropenem* or meropenemvaborbactam* or methicillin* or metronidazole* or mezlocillin* or mitomycin* or moxifloxacin* or nafcillin* or netilmicin* or oritavancin* or oxacillin* or oxytetracycline* or penicillin* or piperacillin* or piperacillin-tazobactam* or piperacillintazobactam* or plazomicin* or plicamycin* or polymyxin* or quinupristin-dalfopristin* or sulfamethoxazole* or sulfisoxazole* or teicoplanin* or telavancin* or temocillin* or ticarcillin* or tigecycline* or tobramycin* or trovafloxacin* or vancomycin* or aciclovir* or acyclovir* or amphotericin* or liposomal-amphotericin* or ambisome* or anidulafungin* or caspofungin* or eravacycline* or famciclovir* or fluconazole* or flucytosine* or foscarnet* or ganciclovir* or isavuconazonium* or isoconazole* or micafungin* or pentamidine* or rifampin* or voriconazole*) or (agents, antiinfective[mesh terms])) or (anti-infective therapy[mesh terms])) or (antibiotic* or anti-biotic* or anti-bacterial* or antibacterial* or antimicrobial* or anti-microbial* or anti-fungal* or anti-viral* or antiviral* or infectious agent*)) and (((((drug delivery system[mesh terms]) or (elastomeric-pump* or balloon-pump* or ball-pump* or portable pump* or accufuser* or lv10*)) or (external infusion pump[mesh terms])) or (infusion pump[mesh terms])) or (elastomer[mesh terms]))) and ((administration, intravenous[mesh terms]) or (intravenous* or parenteral*))",most recent,,"(""amikacin*""[all fields] or ""amoxicillin*""[all fields] or ""amoxycillin*""[all fields] or ""ampicillin*""[all fields] or ""azithromycin*""[all fields] or ""aztreonam*""[all fields] or ""bleomycin*""[all fields] or ""carbenicillin*""[all fields] or ""cefazolin*""[all fields] or ""cefepime*""[all fields] or ""cefiderocol*""[all fields] or ""cefmetazole*""[all fields] or ""cefonicid*""[all fields] or ""cefoperazone*""[all fields] or ""cefotaxime*""[all fields] or ""cefotetan*""[all fields] or ""cefoxitin*""[all fields] or ""ceftaroline*""[all fields] or ""ceftazidime*""[all fields] or ""ceftazidimeavibactam*""[all fields] or ""ceftazidime avibactam*""[all fields] or ""ceftizoxime*""[all fields] or ""ceftolozanetazobactam*""[all fields] or ""ceftolozane tazobactam*""[all fields] or ""ceftriaxone*""[all fields] or ""cefuroxime*""[all fields] or ""cephalothin*""[all fields] or ""cephapirin*""[all fields] or ""chloramphenicol*""[all fields] or ""ciprofloxacin*""[all fields] or ""clarithromycin*""[all fields] or ""clindamycin*""[all fields] or ""cloxacillin*""[all fields] or ""colistimethate*""[all fields] or ""dactinomycin*""[all fields] or ""daptomycin*""[all fields] or ""delafloxacin*""[all fields] or ""doripenem*""[all fields] or ""eravacycline*""[all fields] or ""ertapenem*""[all fields] or ""erythromycin*""[all fields] or ""flucloxacillin*""[all fields] or ""fosfomycin*""[all fields] or ""fusidic*""[all fields] or ""gentamicin*""[all fields] or ""imipenem*""[all fields] or ""imipenem cilastatin	2,311

	relebactam*""[all fields] or ""kanamycin*""[all fields] or ""lefamulin*""[all fields] or ""levofloxacin*""[all fields] or ""lincomycin*""[all fields] or ""linezolid*""[all fields] or ""meropenem*""[all fields] or ""meropenemvaborbactam*""[all fields] or ""methicillin*""[all fields] or ""metronidazole*""[all fields] or ""mezlocillin*""[all fields] or ""mitomycin*""[all fields] or ""moxifloxacin*""[all fields] or ""nafcillin*""[all fields] or ""netilmicin*""[all fields] or ""oritavancin*""[all fields] or ""oxacillin*""[all fields] or ""oxytetracycline*""[all fields] or ""penicillin*""[all fields] or ""piperacillin*""[all fields] or ""piperacillin tazobactam*""[all fields] or ""piperacillintazobactam*""[all fields] or ""plazomicin*""[all fields] or ""plicamycin*""[all fields] or ""polymyxin*""[all fields] or ""quinupristin dalfopristin*""[all fields] or ""sulfamethoxazole*""[all fields] or ""sulfisoxazole*""[all fields] or ""teicoplanin*""[all fields] or ""telavancin*""[all fields] or ""temocillin*""[all fields] or ""ticarcillin*""[all fields] or ""tigecycline*""[all fields] or ""tobramycin*""[all fields] or ""trovafloxacin*""[all fields] or ""vancomycin*""[all fields] or ""aciclovir*""[all fields] or ""acyclovir*""[all fields] or ""amphotericin*""[all fields] or ""liposomal amphotericin*""[all fields] or ""ambisome*""[all fields] or ""anidulafungin*""[all fields] or ""caspofungin*""[all fields] or ""eravacycline*""[all fields] or ""famciclovir*""[all fields] or ""fluconazole*""[all fields] or ""flucytosine*""[all fields] or ""foscarnet*""[all fields] or ""ganciclovir*""[all fields] or ""isavuconazonium*""[all fields] or ""isoconazole*""[all fields] or ""micafungin*""[all fields] or ""pentamidine*""[all fields] or ""rifampin*""[all fields] or ""voriconazole*""[all fields] or ""anti infective agents""[mesh terms] or ((""anti infective agents""[pharmacological action] or ""anti infective agents""[mesh terms] or (""anti infective""[all fields] and ""agents""[all fields]) or ""anti infective agents""[all fields] or (""anti""[all fields] and ""infective""[all fields]) or ""anti infective""[all fields]) and ""therapeutics""[mesh terms]) or (""antibiotic*""[all fields] or ""anti biotic*""[all fields] or ""anti bacterial*""[all fields] or ""antibacterial*""[all fields] or ""antimicrobial*""[all fields] or ""anti microbial*""[all fields] or ""anti fungal*""[all fields] or ""anti viral*""[all fields] or ""antiviral*""[all fields] or ((""infectious""[all fields] or ""infectiousness""[all fields]) and ""agent*""[all fields]))) and (""drug delivery systems""[mesh terms] or (""elastomeric pump*""[all fields] or ""balloon pump*""[all fields] or ""ball pump*""[all fields] or ((""portability""[all fields] or ""portable""[all fields] or ""portables""[all fields]) and ""pump*""[all fields]) or ""accufuser*""[all fields] or ""lv10*""[all fields]) or ""infusion pumps""[mesh terms] or ""infusion pumps""[mesh terms] or ""elastomers""[mesh terms]) and (""infusions, intravenous""[mesh terms] or (""intravenous*""[all fields] or ""parenteral*""[all fields]))"	
17	16 limited to 2021-2022	117

Table S7 – OPENGREY search strategy and results 21st November 2021

	Search Terms	Hits
1	Elastomeric pump* or balloon pump* or ball pump* or portable pump* or accufuser* or lv10* or ntimate* or infusor*	70,935
2	Antiinfective agent* or anti-infective* or infectious agent* or antibiotic*or antifungal agent* or antivirus agent* or amikacin* or amoxicillin* or	1,391

	amoxycillin* or ampicillin* or azithromycin* or aztreonam* or bleomycin* or carbenicillin* or cefazolin* or cefepimecefiderocol* or cefmetazole* or cefonicid* or cefoperazone* or cefotaxime* or cefotetan* or cefoxitin* or ceftaroline* or ceftazidime* or ceftazidimeavibactam*ceftazidime-avibactam* or ceftizoxime* or ceftolozanetazobactam* or ceftolozane-tazobactam* or ceftriaxone* or cefuroxime* or cephalothin* or cephapirin* or chloramphenicol* or ciprofloxacin*clarithromycin* or clindamycin* or cloxacillin* or colistimethate* or dactinomycin* or daptomycin* or delafloxacin* or doripenem* or eravacycline* or ertapenem*erythromycin* or flucloxacillin* or fosfomycin* or fusidic* or gentamicin* or imipenem* or imipenem-cilastatin-relebactam* or kanamycin* or lefamulin* or levofloxacin* or lincomycin* or linezolid* or meropenem* or meropenemvaborbactam* or methicillin* or metronidazole* or mezlocillin* or mitomycin* or moxifloxacin* or nafcillin*netilmicin* or oritavancin* or oxacillin* or oxytetracycline* or penicillin* or piperacillin* or piperacillin-tazobactam* or piperacillintazobactam* or plazomicin* or plicamycin* or polymyxin*quinupristin-dalfopristin* or sulfamethoxazole* or sulfisoxazole* or teicoplanin* or telavancin* or temocillin* or ticarcillin* or tigecycline* or tobramycin* or trovafloxacin* or vancomycin*aciclovir* or acyclovir* or amphotericin* or liposomal-amphotericin* or ambisome* or anidulafungin* or caspofungin* or eravacycline* or famciclovir* or fluconazole*flucytosine* or foscarnet* or ganciclovir* or isavuconazonium* or isoconazole* or micafungin* or pentamidine* or rifampin* or voriconazole*	
3	1 or 2	72337
4	3 and intravenous* or parenteral* or continuous*	46

Table S8 – Google scholar search strategy and results 22nd December 2022

	Search Terns	Hits
1	Elastomeric infusion pumps antimicrobial	2,130 (first 200 included)

Table S9 – BIOSIS search strategy and results 20th January 2023

	Search Terms	Hits
1	Ts=(elastomeric pump* or balloon pump* or ball pump* or portable pump* or accufuser* or lv10* or ntimate* or infusor*)	6,646
2	Ts=(amikacin* or amoxicillin* or amoxycillin* or ampicillin* or azithromycin* or aztreonam* or bleomycin* or carbenicillin* or cefazolin* or cefepimecefiderocol* or cefmetazole* or cefonicid* or cefoperazone* or cefotaxime* or cefotetan* or cefoxitin* or ceftaroline* or ceftazidime* or ceftazidimeavibactam*ceftazidime-avibactam* or ceftizoxime* or ceftolozanetazobactam* or ceftolozane-tazobactam* or ceftriaxone* or cefuroxime* or cephalothin* or cephapirin* or chloramphenicol* or ciprofloxacin*clarithromycin* or clindamycin* or cloxacillin* or colistimethate* or dactinomycin* or daptomycin* or delafloxacin* or doripenem* or eravacycline* or ertapenem*erythromycin* or flucloxacillin* or fosfomycin* or fusidic* or gentamicin* or imipenem* or imipenem-cilastatin-relebactam* or kanamycin* or lefamulin* or levofloxacin*lincomycin* or linezolid* or meropenem* or meropenemvaborbactam* or methicillin* or metronidazole* or mezlocillin* or mitomycin* or moxifloxacin* or nafcillin*netilmicin* or oritavancin* or oxacillin* or oxytetracycline* or penicillin* or piperacillin* or piperacillin-tazobactam* or	378,152

	piperacillintazobactam* or plazomicin* or plicamycin* or polymyxin*quinupristin-dalfopristin* or sulfamethoxazole* or sulfisoxazole* or teicoplanin* or telavancin* or temocillin* or ticarcillin* or tigecycline* or tobramycin* or trovafloxacin* or vancomycin*aciclovir* or acyclovir* or amphotericin* or liposomal-amphotericin* or ambisome* or anidulafungin* or caspofungin* or eravacycline* or famciclovir* or fluconazole*flucytosine* or foscarnet* or ganciclovir* or isavuconazonium* or isoconazole* or micafungin* or pentamidine* or rifampin* or voriconazole*)	
3	Ts=(antiinfective agent* or anti-infective* or infectious agent* or antibiotic*or antifungal agent* or antivirus agent)	536,581
4	3 or 2	767,434
5	1 and 4	112
6	5 between 21 Nov 2021 and 20 Jan 2023	5

Table S10 – Web of Science (WOS) search strategy and results 20th Jan 2023

	Search Terms	Hits
1	All=(elastomeric infusion pump* or infusion pump* elastomeric pump* or balloon pump* or ball pump* or portable pump* or accufuser* or lv10* or intimate* or infusor*)	93,421
2	All=(antiinfective agent* or anti-infective* or infectious agent* or antibiotic*or antifungal agent* or antivirus agent*)	122,322
3	All=(amikacin* or amoxicillin* or amoxycillin* or ampicillin* or azithromycin* or aztreonam* or bleomycin* or carbenicillin* or cefazolin* or cefepimecefiderocol* or cefmetazole* or cefonicid* or cefoperazone* or cefotaxime* or cefotetan* or cefoxitin* or ceftaroline* or ceftazidime* or ceftazidimeavibactam*ceftazidime-avibactam* or ceftizoxime* or ceftolozanetazobactam* or ceftolozane-tazobactam* or ceftriaxone* or cefuroxime* or cephalothin* or cephapirin* or chloramphenicol* or ciprofloxacin*clarithromycin* or clindamycin* or cloxacillin* or colistimethate* or dactinomycin* or daptomycin* or delafloxacin* or doripenem* or eravacycline* or ertapenem*erythromycin* or flucloxacillin* or fosfomycin* or fusidic* or gentamicin* or imipenem* or imipenem-cilastatin-relebactam* or kanamycin* or lefamulin* or levofloxacin* or lincomycin*)	200,685
4	All=(linezolid* or meropenem* or meropenemvaborbactam* or methicillin* or metronidazole* or mezlocillin* or mitomycin* or moxifloxacin* or nafcillin*netilmicin* or oritavancin* or oxacillin* or oxytetracycline* or penicillin* or piperacillin* or piperacillin-tazobactam* or piperacillintazobactam* or plazomicin* or plicamycin* or polymyxin*quinupristin-dalfopristin* or sulfamethoxazole* or sulfisoxazole* or teicoplanin* or telavancin* or temocillin* or ticarcillin* or tigecycline* or tobramycin* or trovafloxacin* or vancomycin*aciclovir* or acyclovir* or amphotericin* or liposomal-amphotericin* or ambisome* or anidulafungin* or caspofungin* or eravacycline* or famciclovir* or fluconazole*flucytosine* or foscarnet* or ganciclovir* or isavuconazonium* or isoconazole* or micafungin* or pentamidine* or rifampin* or voriconazole*)	269,291
5	3 or 4	412,884
6	1 and 2	156

7	1 and 3	143
8	1 and 4	179
9	6 or 7	290
10	9 between 21 Nov 2021 and 20 Jan 2023	18

Table S11 – Google search strategy and results 5th February 2023

	Search Terms	Hits
1	Elastomeric pump antimicrobial intravenous	49700000 (first 100 included)

Table S12 – PRIMSA Checklist

Section and Topic	Item #	Checklist item	Location where item is reported
**TITLE**			
Title	1	Identify the report as a systematic review.	Page 1
**ABSTRACT**			
Abstract	2	See the PRISMA 2020 for Abstracts checklist.	
**INTRODUCTION**			
Rationale	3	Describe the rationale for the review in the context of existing knowledge.	Page 1
Objectives	4	Provide an explicit statement of the objective(s) or question(s) the review addresses.	Page 1
**METHODS**			
Eligibility criteria	5	Specify the inclusion and exclusion criteria for the review and how studies were grouped for the syntheses.	Page 4
Information sources	6	Specify all databases, registers, websites, organisations, reference lists and other sources searched or consulted to identify studies. Specify the date when each source was last searched or consulted.	Page 4/ Appendix A1/ supplementary information
Search strategy	7	Present the full search strategies for all databases, registers and websites, including any filters and limits used.	Appendix A1
Selection process	8	Specify the methods used to decide whether a study met the inclusion criteria of the review, including how many reviewers screened each record and each report retrieved, whether they worked independently, and if applicable, details of automation tools used in the process.	Page 4 / Appendix A1
Data collection process	9	Specify the methods used to collect data from reports, including how many reviewers collected data from each report, whether they worked independently, any processes for obtaining or confirming data from study investigators, and if applicable, details of automation tools used in the process.	Page 4
Data items	10a	List and define all outcomes for which data were sought. Specify whether all results that were compatible with each outcome domain in each study were sought (e.g. for all measures, time points, analyses), and if not, the methods used to decide which results to collect.	Page 4 / supplementary materials
	10b	List and define all other variables for which data were sought (e.g. participant and intervention characteristics, funding sources). Describe any assumptions made about any missing or unclear information.	Page 4 / supplementary materials
Study risk of bias assessment	11	Specify the methods used to assess risk of bias in the included studies, including details of the tool(s) used, how many reviewers assessed each study and whether they worked independently, and if applicable, details of automation tools used in the process.	Page 4 / Appendix B
Effect measures	12	Specify for each outcome the effect measure(s) (e.g. risk ratio, mean difference) used in the synthesis or presentation of results.	Page 4 / Supplementary materials

Section and Topic	Item #	Checklist item	Location where item is reported
Synthesis methods	13a	Describe the processes used to decide which studies were eligible for each synthesis (e.g. tabulating the study intervention characteristics and comparing against the planned groups for each synthesis (item #5)).	Page 4 / Supplementary materials
	13b	Describe any methods required to prepare the data for presentation or synthesis, such as handling of missing summary statistics, or data conversions.	Page 4 / Supplementary materials
	13c	Describe any methods used to tabulate or visually display results of individual studies and syntheses.	Page 4
	13d	Describe any methods used to synthesize results and provide a rationale for the choice(s). If meta-analysis was performed, describe the model(s), method(s) to identify the presence and extent of statistical heterogeneity, and software package(s) used.	Page 4
	13e	Describe any methods used to explore possible causes of heterogeneity among study results (e.g. subgroup analysis, meta-regression).	Page 4
	13f	Describe any sensitivity analyses conducted to assess robustness of the synthesized results.	Page 4
Reporting bias assessment	14	Describe any methods used to assess risk of bias due to missing results in a synthesis (arising from reporting biases).	Page 4 / Appendix B
Certainty assessment	15	Describe any methods used to assess certainty (or confidence) in the body of evidence for an outcome.	Page 3
**RESULTS**			
Study selection	16a	Describe the results of the search and selection process, from the number of records identified in the search to the number of studies included in the review, ideally using a flow diagram.	Page 3
	16b	Cite studies that might appear to meet the inclusion criteria, but which were excluded, and explain why they were excluded.	Page 3
Study characteristics	17	Cite each included study and present its characteristics.	Page 3
Risk of bias in studies	18	Present assessments of risk of bias for each included study.	Page 3
Results of individual studies	19	For all outcomes, present, for each study: (a) summary statistics for each group (where appropriate) and (b) an effect estimate and its precision (e.g. confidence/credible interval), ideally using structured tables or plots.	Supplementary materials – data synthesis / Page 3
Results of syntheses	20a	For each synthesis, briefly summarise the characteristics and risk of bias among contributing studies.	Page 3
	20b	Present results of all statistical syntheses conducted. If meta-analysis was done, present for each the summary estimate and its precision (e.g. confidence/credible interval) and measures of statistical heterogeneity. If comparing groups, describe the direction of the effect.	Supplementary materials – data synthesis / Page 3
	20c	Present results of all investigations of possible causes of heterogeneity among study results.	N

Section and Topic	Item #	Checklist item	Location where item is reported
	20d	Present results of all sensitivity analyses conducted to assess the robustness of the synthesized results.	N
Reporting biases	21	Present assessments of risk of bias due to missing results (arising from reporting biases) for each synthesis assessed.	Supplementary materials – data synthesis / Appendix B
Certainty of evidence	22	Present assessments of certainty (or confidence) in the body of evidence for each outcome assessed.	Page 3
**DISCUSSION**			
Discussion	23a	Provide a general interpretation of the results in the context of other evidence.	Page 3
	23b	Discuss any limitations of the evidence included in the review.	Page 3
	23c	Discuss any limitations of the review processes used.	Page 3
	23d	Discuss implications of the results for practice, policy, and future research.	Page 3
**OTHER INFORMATION**			
Registration and protocol	24a	Provide registration information for the review, including register name and registration number, or state that the review was not registered.	Page 3
	24b	Indicate where the review protocol can be accessed, or state that a protocol was not prepared.	Page 3
	24c	Describe and explain any amendments to information provided at registration or in the protocol.	Page 4
Support	25	Describe sources of financial or non-financial support for the review, and the role of the funders or sponsors in the review.	Page 5
Competing interests	26	Declare any competing interests of review authors.	Page 5
Availability of data, code and other materials	27	Report which of the following are publicly available and where they can be found: template data collection forms; data extracted from included studies; data used for all analyses; analytic code; any other materials used in the review.	Y

From: Page MJ, McKenzie JE, Bossuyt PM, Boutron I, Hoffmann TC, Mulrow CD, et al. The PRISMA 2020 statement: an updated guideline for reporting systematic reviews. BMJ 2021;372:n71. doi: 10.1136/bmj.n71

For more information, visit: http://www.prisma-statement.org/

Table S13 – Systematic review without meta-analysis (SWiM) Checklist

SWiM reporting item	Item description	Page in manuscript where item is reported
1 Grouping studies for synthesis	1a) Provide a description of, and rationale for, the groups used in the synthesis (eg, groupings of populations, interventions, outcomes, study design)	Page 2
	1b) Detail and provide rationale for any changes made subsequent to the protocol in the groups used in the synthesis	Page 4
2 Describe the standardised metric and transformation methods used	Describe the standardised metric for each outcome. Explain why the metric(s) was chosen and describe any methods used to transform the intervention effects, as reported in the study, to the standardised metric, citing any methodological guidance consulted	Page 2
3 Describe the synthesis methods	Describe and justify the methods used to synthesise the effects for each outcome when it was not possible to undertake a meta-analysis of effect estimates	Page 2
4 Criteria used to prioritise results for summary and synthesis	Where applicable, provide the criteria used, with supporting justification, to select the particular studies, or a particular study, for the main synthesis or to draw conclusions from the synthesis (eg, based on study design, risk of bias assessments, directness in relation to the review question)	Page 2 / Page 3
5 Investigation of heterogeneity in reported effects	State the method(s) used to examine heterogeneity in reported effects when it was not possible to undertake a meta-analysis of effect estimates and its extensions to investigate heterogeneity	Page 2
6 Certainty of evidence	Describe the methods used to assess the certainty of the synthesis findings	Page 3
7 Data presentation methods	Describe the graphical and tabular methods used to present the effects (eg, tables, forest plots, harvest plots)	Page 2 and supplementary materials
	Specify key study characteristics (eg, study design, risk of bias) used to order the studies, in the text and any tables or graphs, clearly referencing the studies included	
8 Reporting results	For each comparison and outcome, provide a description of the synthesised findings and the certainty of the findings. Describe the result in language that is consistent with the question the synthesis addresses, and indicate which studies contribute to the synthesis	Page 2 and supplementary materials
9 Limitations of the synthesis	Report the limitations of the synthesis methods used and/or the groupings used in the synthesis and how these affect the conclusions that can be drawn in relation to the original review question	Page 3
